# Alternation in the Glycolipid Transfer Protein Expression Causes Changes in the Cellular Lipidome

**DOI:** 10.1371/journal.pone.0097263

**Published:** 2014-05-13

**Authors:** Matti A. Kjellberg, Anders P. E. Backman, Henna Ohvo-Rekilä, Peter Mattjus

**Affiliations:** Biochemistry, Department of Biosciences, Åbo Akademi University, Turku, Finland; University of Louisville, United States of America

## Abstract

The glycolipid transfer protein (GLTP) catalyzes the binding and transport of glycolipids, but not phospholipids or neutral lipids. With its all-alpha helical fold, it is the founding member for a new superfamily, however its biological role still remains unclear. We have analyzed changes in the HeLa cell lipidome in response to down- and up-regulation of GLTP expression. We used metabolic labeling and thin layer chromatography analysis, complemented with a lipidomics mass spectroscopic approach. HeLa cells were treated with GLTP siRNA or were transiently overexpressing the GLTP gene. We identified eight different lipid classes that changed as a result of the GLTP down- or up-regulation treatments; glucosylceramide, lactosylceramide, globotriaosylceramide, ceramide, sphingomyelin, cholesterol-esters, diacylglycerol and phosphatidylserine. We discovered that the amount of globotriaosylceramide (Gb_3_) was extensively lowered after down-regulation of GLTP. Further, an up-regulation of GLTP caused a substantial increase in both the Gb_3_ and glucosylceramide levels compared to the controls. Total galactosylceramide levels remained unchanged. Both lactosylceramide and ceramide showed small changes, an increase with increasing GLTP and a decrease in the HeLa cell GLTP knockdowns. The cholesterol-esters and diacylglycerol masses increased in cells that had upregulated GLTP protein levels, wheras down-regulation did not affect their amounts. For the glycerophospholipids, phosphatidylserine was the only species that was lower in GLTP overexpressing cells. Phosphatidylethanolamine, phosphatidylglyerol and phosphatidylinositol remained unaltered. A total of 142 lipid species were profiled and quantified using shotgun lipidomics analyses. This work provides for the first time insights into how alternations in the levels of a protein that binds and transfers glycolipids affects the cellular lipid metabolism. We discuss the observed changes in the lipidome and how these relate to GLTP. We suggest, that GLTP not only could be a significant player in cellular sphingolipid metabolism, but also could have a much broader role in the overall lipid metabolism.

## Introduction

Previous studies on the biological function of the glycolipid transfer protein (GLTP) have been focusing on events related to its lipid substrate class, the glycosphingolipids (GSLs) [1]–[7]. Little is known how other lipid classes are affected in cells that have altered GLTP expression. The glycolipid transfer protein is a cytosolic protein [1] that catalyzes the transport of both sphingoid and glycerol based glycolipids in vitro [8], [9]. GLTP does not transfer phospholipids, sphingomyelin (SM) or neutral lipids [10]. GLTP with its all-alpha helical fold is the founding member for a new protein superfamily in eukaryotes [11]. GLTP is found in a broad range of species, fungi, red algae, plants and mammals [12]. Several homologues to mammalian GLTP have been found in various species [12]–[15], including the human four-phosphate adaptor protein, FAPP2 that contains a GLTP-motif, and has been shown to mediate the transfer of glucosylceramide (GlcCer) from early Golgi to distal Golgi compartments [5], [15], [16].

In a previous study we analyzed de novo GSL changes in cells overexpressing GLTP (transiently transfected) using ^3^H-sphinganine metabolic labeling [1]. The results showed a significant increase in the de novo synthesis of GlcCer and a decrease in the SM synthesis. However, we did not detect any changes in the sphingolipid synthesis in GLTP-knockdown (RNA interference) cells compared to control cells [1]. In this work we have extended the analysis to also include other lipid classes. Moreover we have used FACS-sorted cells to be able to also detect even small changes that are often missed, if the transfection efficiency is not very effective. GLTP appears to respond to changes in the amount of newly synthesized GlcCer [17]. In another recent study we found that GLTP expression, both at the mRNA and protein levels, is elevated in cells that accumulate GlcCer, generated by brefeldin A treatments [17]. Also an 80% loss of GlcCer, caused by glucosylceramide synthase knockdown, resulted in a significant reduction in the expression of GLTP [17].

In the present study we used both metabolic labeling with TLC analysis and a lipidomic approach. The metabolic labeling of sphingolipids, phospholipids and sterols were initially used and complemented with a more detailed analysis of the individual lipid species by the MS approach. All in all, we investigated the changes in 15 different lipid classes and in 142 molecular lipid profiles of HeLa cells as a function of GLTP down- or up-regulation. The MS analysis was done by Zora Biosciences (Espoo, Finland) utilizing a shotgun lipidomics approach employing a hybrid triple quadrupole/linear ion trap mass spectrometer. We have discovered that the globotriaosylceramide level (Gb_3_) showed a strong decrease after GLTP siRNA treatment, and that the up-regulation of GLTP caused an increase in both the Gb_3_ and GlcCer levels compared to the control HeLa cells. The only glycerophospholipid that we detected changes in was phosphatidylserine (PS), whereas phosphatidylethanolamine (PE), phosphatidylglyerol (PG) and phosphatidylinositol (PI) levels remained unaltered.

## Materials and Methods

### Materials & Cell Culture

All chemical reagents were of analytical grade or higher. HeLa cervical carcinoma cells (ATCC CCL-2, LGC Standards) were cultured in 100 mm dishes (Greiner Bio-One GmbH, Germany) in Dulbecco’s Modified Eagle’s medium, Sigma (St. Louis, MO, USA) supplemented with penicillin (50 U/ml), streptomycin (50 U/ml), 4 mM L-glutamine and 10% fetal calf serum Sigma (St. Louis, MO, USA) prior to the experiments. The human GLTP (NM_016433.3) gene was cloned into a pcDNA3.1(+)-vector, Invitrogen (Carlsbad, CA, USA), between the cloning sites BamH1/EcoR1 (hGLTP) [1]. The empty green fluorescent protein pEGFPN1 vector, Clontech (Palo Alto, CA, USA) was used for fluorescence-activated cell sorting (FACS) transfection efficiency determination and sorting purposes of the GLTP overexpressing cells. Human GLTP Stealth RNAi and a Stealth RNAi negative control were obtained from Invitrogen (Carlsbad, CA, USA). Lipids standards for the TLC analysis where from Avanti Polar Lipids (Alabaster, USA) or Matreya LLC (Pleasant Gap, USA). The different lipid species of phosphatidylcholine (PC), phosphatidylethanolamine (PE), phosphatidylserine (PS), phosphatidylinositol (PI), phosphatidylglycerol (PG) and diacylglycerol (DAG) are listed with the two fatty acyl groups separated with a hyphen, e.g. PC 16∶0–18∶1. The ether-linked phospholipids are shown as PC O (alkyl), PE O (alkyl) and cholesteryl esters as CE. The acyl chains of ether-linked lipids and the N-linked acyl chains for sphingomyelin (SM), ceramide (Cer) and glycosphingolipids (GSLs) are shown after the slash. All samples, including the controls were cultured in the presence of the same amount of media and FCS and we assume that the small amount of FCS in the culture media is not affecting the cellular lipid metabolism [18].

### Stealth RNAi Knockdown Experiments

The specific siRNA sequences (Invitrogen) were named #76; sense AUACAUUUCUUUCUCCACCUCCAGG and anti-sense CCUGGAGGUGGAGAAAGAAAUGUAU, #77; sense strand, ACUUAUAGGGUGCUGCGUACAGUGC and anti-sense strand, GCACUGUACGCAGCACCCUAUAAGU and #78; sense UUAAGCUCAGCGUUCAUCUGGGUGU and anti-sense ACACCCAGAUGAACGCUGAGCUUAA.

For the metabolic labeling and TLC analysis experiments a mixture of both expression constructs #76 and #78 of GLTP siRNA were used. The efficiency of the different siRNA constructs was analyzed both by qPCR and Western blot analysis. The qPCR analysis was performed as described previously [17]. For the MS analysis experiments HeLa cells, passage 8, were plated at 50% confluence in ten 60 mm dishes and transiently transfected with 75 pmol of GLTP siRNAs #77 with Lipofectamine 2000 (Invitrogen) according to the manufacturer’s instructions for 24 h. Ten different transfections were performed for each sample. After 24 h the cells were split (1/3) cultured for another 48 h in 100 mm dishes in DMEM medium supplemented with 2% fetal calf serum without penicillin/streptomycin and finally harvested for subsequent sorting. After FACS analysis the cells were counted, washed with PBS and aliquoted into two vials containing 5×10^6^ cells each, dried and flash frozen in liquid nitrogen and stored at −80°C awaiting lipidomics analysis. Zora Biosciences Ltd. (Espoo, Finland) analyzed the two samples once. Fluorescein isothiocyanate labeled (FITC) dsRNA oligomers, Block-iT Fluorescence Oligo (Invitrogen, Carlsbad, CA) were used to determine siRNA transfection efficiencies. The oligos were co-transfected with the GLTP siRNA for FACS, enabling us to harvest transfected HeLa cells for subsequent lipidomics analysis. Two Stealth RNAi negative universal control (UC) samples were also generated in parallel with the siRNA GLTP samples, and the two different samples were also analyzed once by Zora Biosciences.

### GLTP Overexpression

5×10^6^ HeLa cells (passage 10) were transiently transfected using a Gene Pulser II RF Module, Bio-Rad (Hercules, CA, USA) electroporator (30 ms), with 5 parallel samples with 10 µg pcDNA-GLTP or 10 µg control pcDNA 3.1(+)-vector, both co-transfected with 10 µg of the pEGFP vector. This was repeated twice. The pEGFP was used for FACS transfection efficiency determination and sorting purposes. After electroporation the cells were then incubated in 100 mm dishes in DMEM supplemented with 2% fetal calf serum. After 48 h the cells were trypsinated, washed and sorted. The pEGFP positive cells (between 30–40%) were sorted and pooled to generate sufficient number (5×10^6^) of cells for one MS samples. This sample was analyzed once. One pcDNA 3.1(+)-vector mock control sample was also generated and analyzed once.

### ^3^H-sphinganine and ^3^H-acetate Labeling of Cells, Subsequent Treatments and Lipid Extraction

HeLa cells to be used for GLTP overexpression experiments were cultured to 95% confluency in 100 mm cell dishes (9–10×10^6^. cells), and trypsinized. The cells were transfected using a Bio-Rad Gene Pulser II electroporation system, as described above, and transferred to 35 mm cell dishes in triplicates. An empty pcDNA 3.1(+)-vector was used as a control. The cells were left to overexpress the protein for 48 h after which they were harvested for further analysis. Only trace doses of precursor lipids were used. This does not alter the normal lipid metabolism of the cells. We added ^3^H-acetate (5.3 µCi per milliliter) 6 h before harvesting the cells that were used to analyze the phospholipid expressions. Similarly, 1.0 µCi/ml of ^3^H-sphinganine was used for the sphingolipid analysis experiments. HeLa cells to be treated with GLTP siRNA were grown to 50% confluency in 35 mm cell plates, after which a knockdown was performed, using Lipofectamine 2000 according to the manufacturer’s instruction. 48 h after beginning the KD, we added 5.3 µCi/ml ^3^H-acetate for 6 h, after which the cells were harvested for further analysis of phospholipids. Similarly, 1.0 µCi/ml of ^3^H-sphinganine was used for the sphingolipid analysis experiments. The cells used for the labeling experiments were not sorted. Treated cells were washed three times in PBS. Total lipids were extracted directly from the culture dishes using hexane:2-propanol (3∶2, v/v) and the extract was dried with nitrogen. The lipids were re-dissolved in hexane:2-propanol and analyzed on high performance thin-layer chromatography (HPTLC) silica plates (Whatman, UK) described below.

### Identification of GSL Species with TLC

For GlcCer, GalCer and LacCer separation the solvent system chloroform:methanol:acetone:acetic acid:water (10∶2:4∶2:1) was used. For Gb_3_ separation the solvent system 45∶55:10, chloroform:methanol:0.2% CaCl_2_ (in H_2_O) was used. The GSL analysis was done using standards, run in parallel with the samples. Lipid migration was visualized using orcinol spray (0.2% orcinol in a 50% H_2_SO_4_ solution) and heating the plate on 120°C for 5 minutes. The lipid spots were scraped into Optiphase ‘Hi phase’ scintillation liquid (PerkinElmer-Wallac, Turku, Finland) and the radioactivity was measured using a liquid scintillation counter, 1216 Rackbeta (PerkinElmer-Wallac, Turku, Finland). After lipid extraction, the total cellular proteins were extracted with 0.1 M NaOH and the protein content was analyzed with the Lowry method [19]. Counts per minute obtained for the various experiments were normalized to total cellular protein. The results are displayed as the ^3^H-sphinganine incorporation into various lipid normalized to the counts per minutes for the control cells.

### Identification of Phospholipids with TLC

Around 35% of the total extracted lipids were applied to the HPTLC-plate, with 10 nmol of carrier lipids to help locate the correct bands [20]. The plates were developed chromatographically in an upright tank, using chloroform:methanol:acetic acid:water; 50∶30:8∶3 as the solvent until the elution front was approximately 1 cm from the edge of the plate. The plates were then stained using iodine vapor, spots marked with a pencil and scraped into scintillation vials. The results are displayed as the ^3^H-acetate incorporation into the phospholipids, normalized to the counts per minutes for the control cells.

### Flow Cytometry and Cell Sorting

The FITC (siRNA) and GFP (GLTP overexpression) positive cells were sorted from the untransfected cells to ensure a reliable lipidomics analysis. The flow cytometry was performed at the Cell Imaging Core facility at the Turku Centre for Biotechnology, Turku, Finland.

### Western Blot

GLTP expression levels were analyzed by Western blotting. The cells were redissolved in a lysis buffer (50 mM NaH_2_PO_4_, 300 mM NaCl, 10 mM imidazole, 0.05% Tween-20, 0.5 mM PMSF, 1×Protease inhibitor cocktail (Sigma), 1 mM dithiotreithol, pH 8.0). The cells were sonicated using a Branson 250 probe sonifier (Emerson Industrial Automation, St. Louis, MO, USA), and the lysate protein concentration was determined using the method of Lowry [19]. The cell lysates were separated on SDS–PAGE and transferred onto a PVDF membrane. Immunoblots were carried out using antibodies against GLTP [1], [21] and β-actin as a loading control. The rabbit anti-beta-actin antibody was from Rockland Immunochemicals (Gilbertsville, PA, USA) and the secondary peroxidase conjugated rabbit anti-goat antibody was from Thermo Scientific (Waltham, MA, USA). The detected proteins were visualized with the ECL chemiluminescence system (SuperSignal West Femto, Thermo Scientific) using X-ray film (Fujifilm, Tokyo, Japan).

### Cell Sample Handling, Storage and Lipid Extraction

The extraction of the HeLa cell samples and the lipidomics analyses was conducted by Zora Biosciences Oy (Espoo, Finland) according to their standard operating procedures. The dry flash frozen HeLa cell samples with known cell count (5×10^6^) were delivered and stored at −80°C prior to analysis. The samples were thawed in a chilly environment upon starting the extraction. Briefly, the dry flash frozen HeLa cell samples were thawed in a chilly environment and re-suspended in ice-cold PBS prior to extraction. Cells were counted and all measurements were normalized to the cell number. A volume corresponding to 0.5 million cells was used for lipid extraction. Lipids were extracted using a modified Folch extraction procedure [22] performed on a Hamilton Microlab Star robot. Known amounts of deuterium-labeled or heptadecanoyl-based synthetic internal standards of SM, LPC, PC, PE, PS, PG, PA, DAG, CE, Cer, GlcCer, LacCer, and Gb_3_, were added and used for quantification of the endogenous lipid species as described [23]–[25]. Following lipid extraction, samples were reconstituted in 1∶2 (v/v) chloroform/methanol and stored at −20°C prior to MS analysis.

### MS Analyses

The species of all phospholipids, SM, DAG and CE were analyzed by shotgun analysis on a hybrid triple quadrupole/linear ion trap mass spectrometer (QTRAP 5500, AB SCIEX, MA) equipped with a robotic nanoflow ion source (NanoMate HD, Advion Biosciences, NY) [26]. These analyses were performed using both positive and negative ion modes using multiple precursor ion scanning (MPIS) and neutral loss (NL) based methods [27], [28], whereas CEs were analyzed in positive ion mode [29]. Sphingolipids were analyzed by reverse phase ultra-high pressure liquid chromatography (UHPLC) as previously described [30] using an Acquity BEH C18, 2.1×50 mm column with a particle size of 1.7 µm (Waters, Milford, MA) coupled to a hybrid triple quadrupole/linear ion trap mass spectrometer (QTRAP 5500, AB SCIEX, MA). A 25 min gradient using 10 mM ammonium acetate in water with 0.1% (v/v) formic acid (mobile phase A) and 10 mM ammonium acetate in 4∶3 (v/v) acetonitrile:2-propanol containing 0.1% (v/v) formic acid (mobile phase B) was used. Quantification of sphingolipids was performed using multiple reaction monitoring. Lipidomic data is based on the analysis of each detected lipid class with one technical replicate for each cell sample. The samples, CTRL cells, GLTP siRNA and GLTP OE cells were in duplicates. The MS lipidomic analyses were performed in the Zora Biosciences laboratory that works according to Good Laboratory Practice, and the published validation data show less than 15% variation for most lipid species [31].

### Lipidomics Data Processing

The MS data files were processed as previously described [24] using LipidView and MultiQuant software for producing a list of lipid names and peak areas. A stringent cutoff was applied for separating background noise from actual lipid peaks. Masses and counts of detected peaks were converted into a list of corresponding lipid names. Lipids were normalized to their respective internal standard [24] and the concentrations of molecular lipids are presented as pmol/500 000 cells.

### Quality Control

Quality control samples were utilized to monitor the overall quality of the lipid extraction and mass spectrometry analyses [23] mainly to remove technical outliers and lipid species that were detected below the limit of quantification.

### Statistical Significance

The statistical significance compared to the respective controls is indicated with asterisks. One asterisk (*) p<0.05, two asterisks (**) p<0.01 and three asterisks (***) p<0.005 indicate the statistical significance compared to the controls.

## Results

### GLTP Expression Levels after Down- and Up-regulation

qPCR analysis clearly showed a reduction of the GLTP gene expression as a function of time in the HeLa small interfering RNA (siRNA) transfected cells (Fig. S1A). All three siRNA sequences, termed #76, #77 and #78 used in this work were compared and normalized to the siRNA universal control (UC), Fig. S1A. Western blot analysis was also performed on the cell samples that were treated with the different siRNA constructs. The blots for normal control cells, UC, siRNA #76, #77, #78 and beta-actin are shown in Fig. S1B. Based on these control experiments, we found that the optimal GLTP siRNA concentrations to treat the HeLa cells were 75 nM, and gene sequence construct #77 was chosen for the MS lipidomics analysis. A mixture of both constructs #76 and #78 was used for the metabolic labeling experiments. Using #77 siRNA GLTP we achieved a yield of 94.0% FITC-positive cells for the knockdown transfection, and 90.5% of the UC transiently transfected cells had taken up the FITC-labeled dsRNA oligos. Cells for the metabolic labeling experiments were not sorted.

We also analyzed the GLTP levels in HeLa total cell lysates with GLTP up-regulated gene expression. Twenty-four hours after HeLa cells were transiently transfected with electroporation with the pcDNA-GLTP construct a very strong expression of GLTP was observed, with a slow decrease as a function of time (Fig. S2A, lower blot). Due to the low expression of endogenous GLTP the band in the control lane (C) is not visible in this blot (30 µg, loading, total cell lysate). Beta-actin was used as a loading control (Fig. S2A, upper blot). HeLa cells that were sorted and harvested for the MS lipidomics analysis were analyzed for expression of GLTP. In the left blot we show the endogenous expression of GLTP (lane 1) and the reduced GLTP expression in HeLa cells with GLTP knockdown, by siRNA (#77 siRNA GLTP gene construct), lane 2. A total of 80 µg total cell lysate was loaded, and beta-actin was used as the loading control, upper blot. The right blot shows the amount of GLTP in HeLa cells with GLTP overexpression (lane 4), and an invisible endogenous GLTP band in lane 3, due to the loading amount of just 10 µg total cell lysate, to avoid overloading of the OE sample. Beta-actin was used as a loading control (Fig. S2B, upper blots), lanes 1 and 2 were loaded with 80 µg of total cell lysate and lanes 3 and 4 with 10 µg of total cell lysate. No signs of abnormal morphological changes or cell death were observed after the down- and up-regulation treatments of the HeLa cells. The cells were routinely visualized during the whole culturing process.

### Precursor Incorporation into GlcCer, GalCer, LacCer and Gb_3_ and TLC Analysis

**Figure 1:**
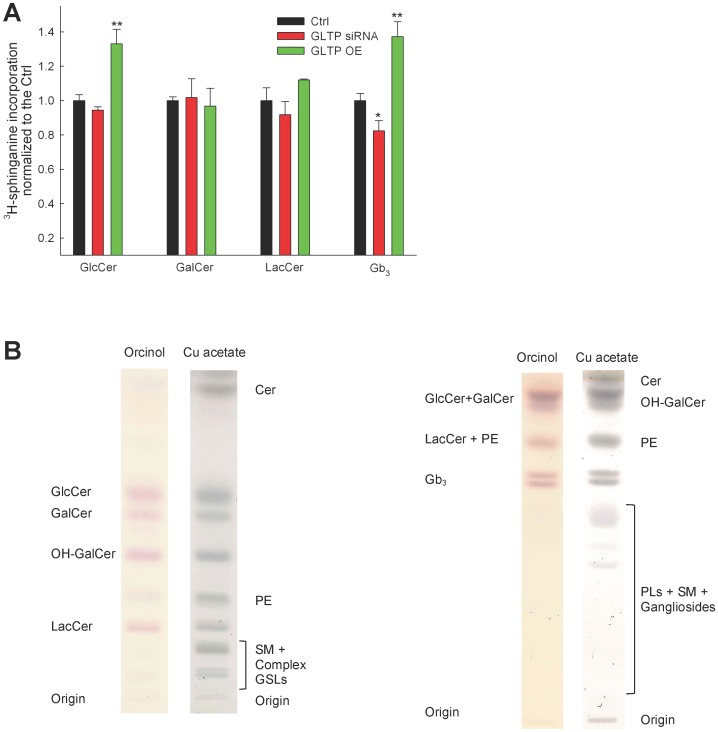
TLC analysis of metabolically labeled GSLs. (**A**) Metabolic labeling of HeLa cells, normal (black), GLTP siRNA (red) and overexpression of GLTP (green). ^3^H-sphinganine incorporation into the sphingolipids was analyzed using TLC, and a mixture of both gene sequence #76 and #78 of the siRNA GLTP constructs were used. The TLC data for the incorporation of the radiolabeled ^3^H-sphinganine are from at least three different experiments, and the results are normalized to the controls. (**B**) Representative TLC plates illustrating a typical separation of the analyzed lipids. The plates were first stained with the carbohydrate specific orcinol, followed by 3% Cu acetate charring to visualize all lipids. Left plate, shows the effective separation of GlcCer, GalCer and LacCer using the solvent system 10∶2:4∶2:1 chloroform:methanol:acetone:acetic acid:H_2_O (v/v). Right plate, for effective Gb_3_ separation from other GSLs we used the 45∶55:10 chloroform:methanol:0.2% CaCl_2_ (in H_2_O) (v/v) solvent system. The statistical significance compared to the respective controls is indicated with asterisks. One asterisk (*) p<0.05 and two asterisks (**) p<0.01 indicate the statistical significance compared to the controls.

Previously we found using the same labeling technique that HeLa cells overexpressing GLTP showed an increase in the de novo GlcCer synthesis [1]. In the previous work the siRNA GLTP gene construct #77 was used, the same as we used in this work for the MS lipidomics approach. For the metabolic labeling experiments we used two gene sequence constructs together (#76 & #78). We analyzed, using ^3^H-sphinganine incorporation, the levels of GlcCer and GalCer, because these two were not distinguishable using the MS protocol to be used in this study. Like previously, the levels of GalCer are at the same level as the control cells, regardless of the regulation of GLTP expression (Fig. 1A). The differences in the LacCer levels are not as pronounced in the metabolic labeling experiments as in the MS analysis data. However it appears the LacCer increases with increased GLTP and vice versa (Fig. 1A). The analysis by TLC of the total de novo Gb_3_ amount after ^3^H-sphinganine incorporation in HeLa cells show a significant increase in the levels of Gb_3_ compared to the levels in the controls. The GLTP siRNA cells also show a significant decrease in the incorporation of the radiolabeled precursor (Fig. 1A). Representative TLC plates showing the separation of the analyzed lipids, Fig. 1B. Right plate, shows the separation used for GlcCer, GalCer and LacCer using the solvent system 10∶2:4∶2:1 chloroform:methanol:acetone:acetic acid:H_2_O (w/w). Left plate, used for Gb_3,_ were the 45∶55:10 chloroform:methanol:0.2% CaCl_2_ (in H_2_O) solvent system was used. During the chromatography Gb_3_ separates into two bands. Heterogeneity in the composition of the fatty acid moieties and the Gb_3_ head group structure (its isomer isoglobo-Gb_3_) results in double bands [32], [33]. Both bands were scraped and analyzed and termed Gb_3_.

### Precursor Incorporation into SM, PC, and PE and their TLC Analysis

**Figure 2:**
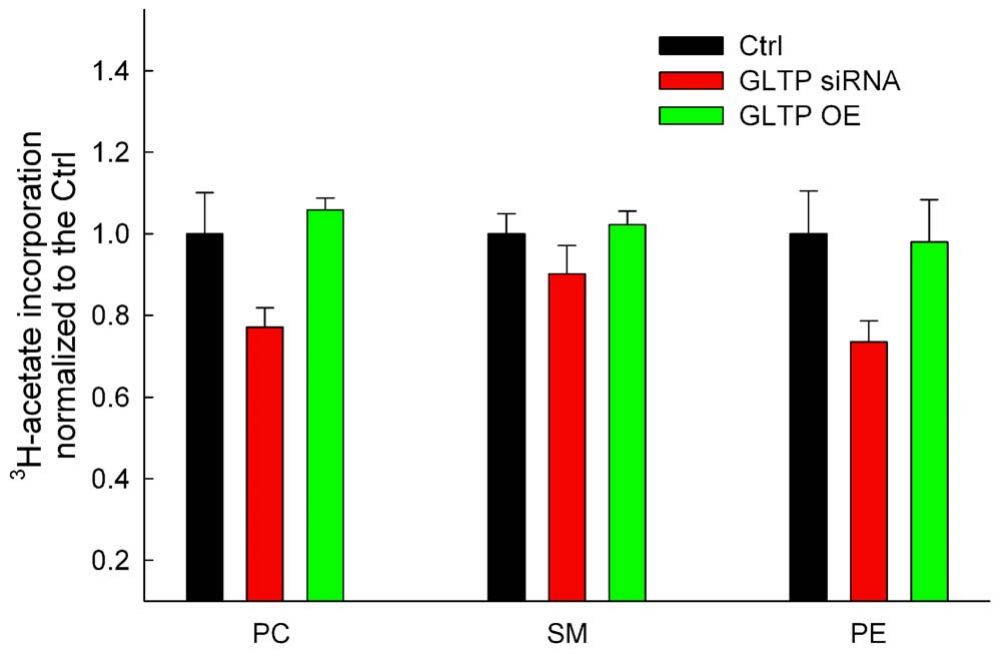
Analysis of metabolically labeled PC, SM and PE after alternation in the GLTP expression levels. Three phospholipid fractions were analyzed using ^3^H-acetate incorporation of HeLa cells with different expression levels of GLTP. The TLC data for the incorporation of the radiolabeled ^3^H-acetate are from at least three different experiments, and the results were normalized to the controls. The statistical significance compared to the respective controls is indicated with asterisks. Two asterisks (**) p<0.01 indicate the statistical significance compared to the controls.

With the use of ^3^H-acetate incorporation we could detect a small decrease in all phospholipid levels for GLTP siRNA treated cells (Fig. 2, red bars). No significant deviations in the phospholipid profiles were observed in the GLTP overexpressing cells (Fig. 2, green bars). The solvent system for the separation of the phospholipids was chloroform:methanol:acetic acid:H_2_O (50∶30:8∶3) (v/v) [34].

### MS Analysis of the Changes in GSL Metabolism after Down- and Up-regulation of GLTP

To get a broader and more detailed picture of the effects the GLTP had on the HeLa cell lipid profile we performed a MS lipidomics analysis. The cells where grown under the same conditions as for the metabolic labeling experiments, with the exception that the GLTP siRNA gene sequence #77 constructs was used. The overexpressing HeLa cells were similar as to the labeling experiments. Cells for the MS analysis were sorted as described in the Material and Methods section.

### GlcCer/GalCer

**Figure 3:**
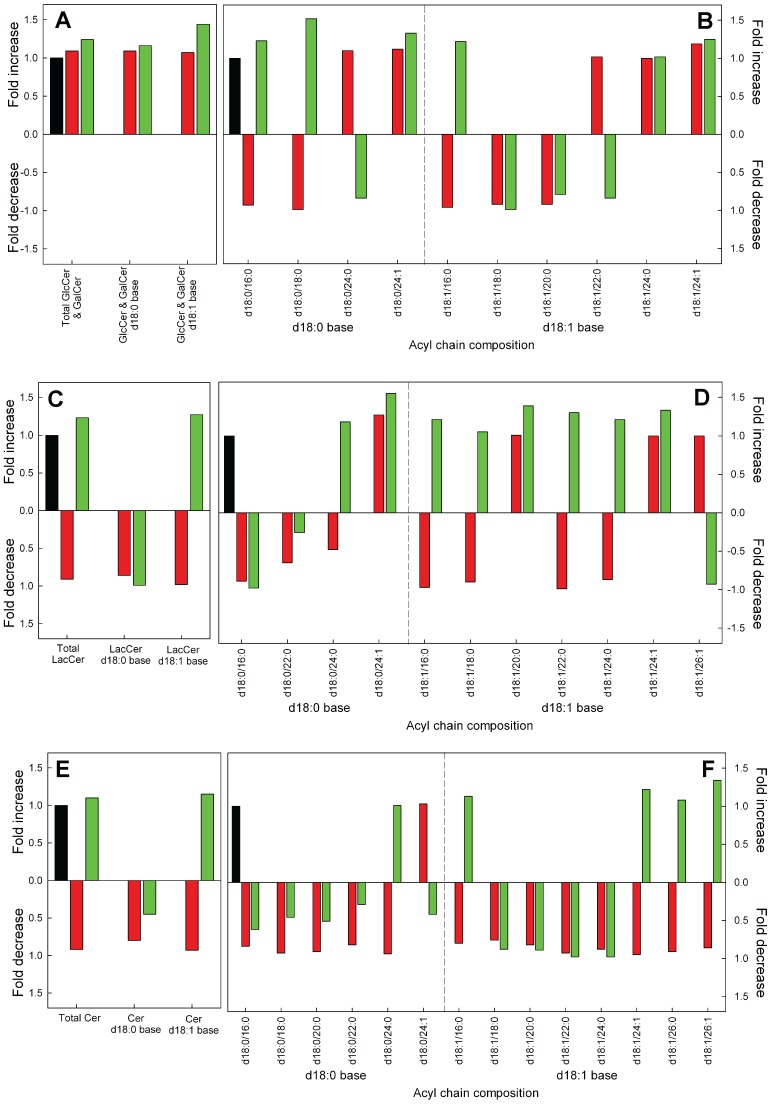
Relative change in GlcCer/Galcer, LacCer and Cer after knockdown or overexpression of GLTP in HeLa cells normalized to control cells. (**A**) Changes in the total masses of GlcCer/GalCer and GlcCer/GalCer with d18∶0 and d18∶1 base respectively. (**B**) Changes in the GlcCer/GalCer species after siRNA or OE treatments, compared to the control. (**C**) Changes in the total masses of LacCer and LacCer with d18∶0 and d18∶1 base respectively and (**D**) changes in the LacCer species. (**E**) Changes in the total masses of Cer and Cer with d18∶0 and D18∶1 base respectively, with (**F**) showing the changes in the Cer species. The data for siRNA GLTP (red bars) and GLTP OE (green bars) were normalized to the levels in the mock control cells (black bars). The abbreviations for the lipid classes are given in “Materials and Methods”. For clarity, minor species are not presented in the graph. For full list of species and quantitative data with SD, see Table S1).

In the lipidomics analysis, on a mass level, there is a small increase in the total cerebrosides for the GLTP OE sample (Fig. 3A), and a larger increase in the total saturated sphinganine d18∶1 base cerebrosides (Fig. 3A). This is probably due the higher levels of GlcCer seen in the metabolic labeling experiments (Fig. 1A). It is important to recall that this MS analysis is for both GlcCer/GalCer. See Table S1 for quantitative data with SD. For clarity, minor species and the SD have been left out. The molecular changes in the acyl chain compositions are shown in Fig. 3B.

### LacCer

The total LacCer amounts did not change significantly in the cells that have up- or down-regulated GLTP (Fig. 3C), however the treated cells had deviating levels of LacCer compared to the control cells. For the LacCer d18∶0 sphinganine base species 22∶0, both siRNA and OE, and the 24∶0 N-linked acyl chain d18∶0 base for the siRNA sample is much lower that the control (Fig. 3D).

### Cer

The total amount of the sphingolipid precursor Cer did not differ significantly from the control cells, however the d18∶0 sphinganine-based Cers in the GLTP siRNA cells were 25% lower (Fig. 3E) and the GLTP OE cells had a reduction of more than 50% compared to the controls (Fig. 3E). It should be noted that the amount of d18∶0 base Cers in the HeLa cells were only one-tenth to that of the d18∶1 base, explaining the only minor differences in the total Cer levels (Table S1).

### Gb_3_

**Figure 4:**
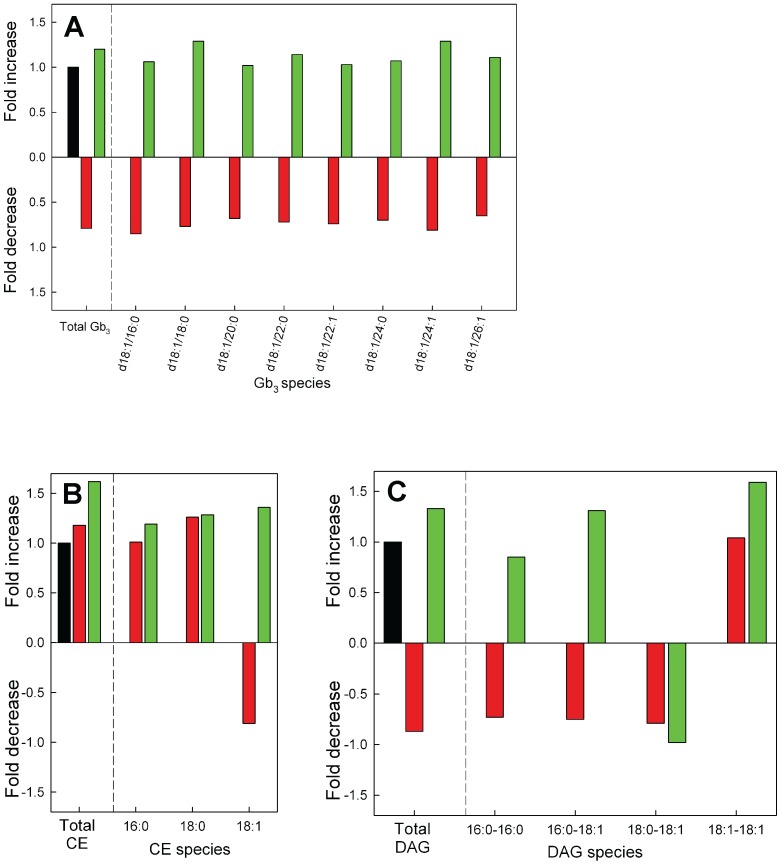
Relative change in Gb_3_, CE and DAG after knockdown or overexpression of GLTP in HeLa cells compared to control cells. (**A**) Changes in total (left side) and molecular species of Gb_3_ in HeLa cells subjected to knockdown (red bars) or overexpression (green bars) of the GLTP gene normalized to the levels in the mock control cells (black bars). The Gb_3_ level for the GLTP siRNA and OE sample is significantly different (p<0.01) than the control sample. (**B**) Relative changes in the total and molecular species of CE’s and (**D**) DAG’s as a function of GLTP expression levels. The abbreviations for the lipid classes are given in “Materials and Methods”. For clarity, minor species are not presented in the graph. For full list of species and quantitative data with SD, see Table S2.

The completely new finding that the amount of Gb_3_ decreased with in the GLTP siRNA treated cells can also be seen in the MS analysis (Fig. 4A, red bars). It should be noted that the amount of Gb_3_ in HeLa cells was 4-fold higher than GlcCer and GalCer together, and 6-fold higher compared to LacCer (Table S2). This is in well agreement with previous reports regarding the balance between Gb_3_ and monohexosylceramides in HeLa cells. [6], [35]. The decrease in the Gb_3_ is in line with the data seen in the FAPP2 knockdown HeLa cells in the very resent study by D’Angelo and co-workers [5]. It appears that all molecular Gb_3_ lipids that were analyzed were lower (Fig. 4A). The amounts of Gb_3_ in the GLTP OE cells showed a small increase compared to the control cells (Fig. 4A, green bar).

### Cholesterol Esters and Diacylglycerol Changes after Up-regulation of GLTP Expression

After knockdown of GLTP we did see a slight but not significant increase for the total CE’s and a small decrease in the DAG amounts (Fig. 4B & 4C, red bars). The levels of CE and DAG on the other hand increased significantly in the HeLa cells overexpressing GLTP (Fig. 4B & 4C, green bars). The quantitative data for the molecular CE’s and the DAGs are presented in Table S2.

### Sphingomyelin and Phosphatidylcholine Levels after Regulation of GLTP Expression

**Figure 5:**
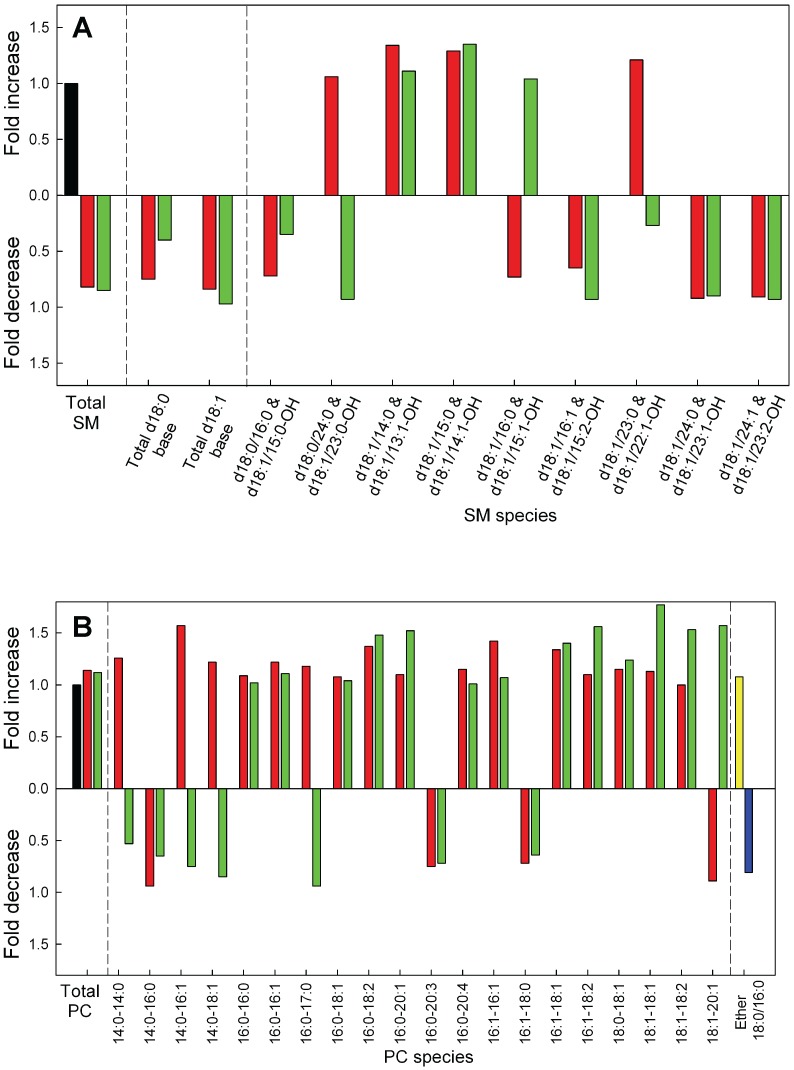
Relative changes in SM, PC and ether-PC after knockdown or overexpression of GLTP in HeLa cells compared to control cells. The relative changes in the masses of (**A**) SM and (**B**) PC in HeLa cells subjected to knockdown (red bars) or overexpression (green bars) of the GLTP gene normalized to the levels in the mock control cells (black bars). The changes for the ether linked PCs is on the far right, yellow represent the GLTP siRNA sample and the blue bar the GLTP OE sample. The abbreviations for the lipid classes are given in “Materials and Methods”. For clarity, minor species are not presented in the graph. For full list of species and quantitative data with SD, see Table S3.

Both in the down- and up-regulated GLTP HeLa cells we found in the MS analysis that the total SM mass decreased compared to the control cells (Fig. 5A). This is comparable to our previous finding that an up-regulation of GLTP causes a decrease in de novo SM synthesis using the metabolic labeling [1]. However we detected a small decrease in the de novo synthesis of SM in the GLTP knockdown cells using ^3^H-acetate incorporation (Fig. 2). The data for the individual molecular lipids of SM is presented in the Table S3. The levels of total PC in the siRNA or GLTP overexpressing cells did not differ significantly from the control HeLa cells (Fig. 5B). The data for the individual molecular lipids of PC is presented in the Table S3. The ether-linked PC 18∶0/16∶0 species was also analyzed with MS but we could not detect any significant changes (Fig. 5B, yellow and blue bars).

### Changes in the PE, PS, PI and PG Metabolism after Down- and Up-regulation of GLTP Expression in HeLa Cells

**Figure 6:**
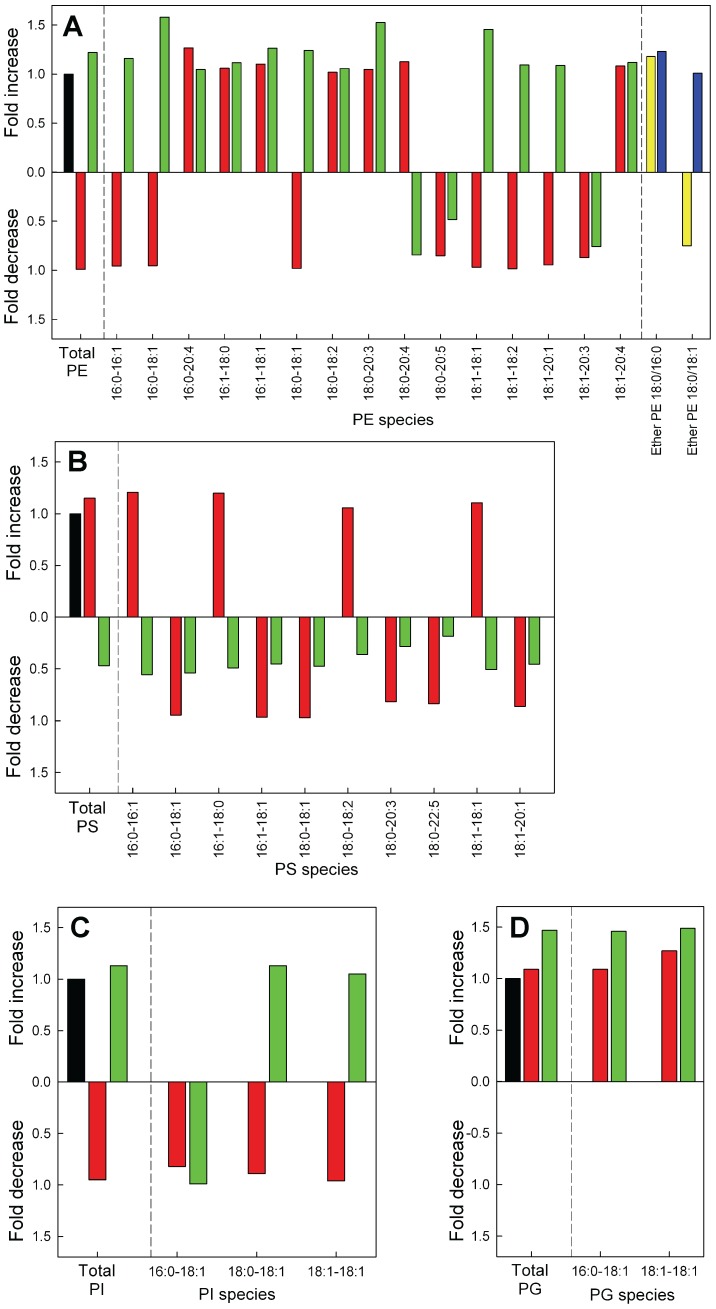
Relative changes in phosphoglycerides after knockdown or overexpression of GLTP in HeLa cells compared to control cells. Changes in the total masses and the molecular species of (**A**) PE, (**B**) PS, (**C**) PI and (**D**) PG in HeLa cells subjected to knockdown (red bars) or overexpression (green bars) of the GLTP gene normalized to the levels in the mock control cells (black bars). The abbreviations for the lipid classes are given in “Materials and Methods”. For clarity, minor species are not presented in the graph. For full list of species and quantitative data with SD, see Table S4.

MS analysis of the total amounts of PE in the treated HeLa cells did not significantly change (Fig. 6A, Table S4). Quantitative analysis shows that PS was significantly reduced in the GLTP overexpressing cells, with a decrease of 50% (Fig. 6B, green bar). The GLTP siRNA cells did show a small increase in the PS mass, however not significant (Fig. 6B, red bar). The distribution of the hydrocarbon chains in PS of HeLa cells shows clearly that all acyl chain species decrease in the GLTP overexpressing HeLa cells (Fig. 6B, green bars). The amount of both PG and PI for the two treated HeLa cell types did not show any significant difference compared to the control cells (Fig. 6C & 6D). The molecular species level changes for PG and PI are shown in Table S4. The ether-linked PE (Table S4) species was also analyzed but we could not detect any significant changes (Fig. 6A, yellow and blue bars).

### The Total Lipid Levels and the Distribution of the Amide-linked Acyl Chains in the Sphingolipids in HeLa Cells

**Figure 7:**
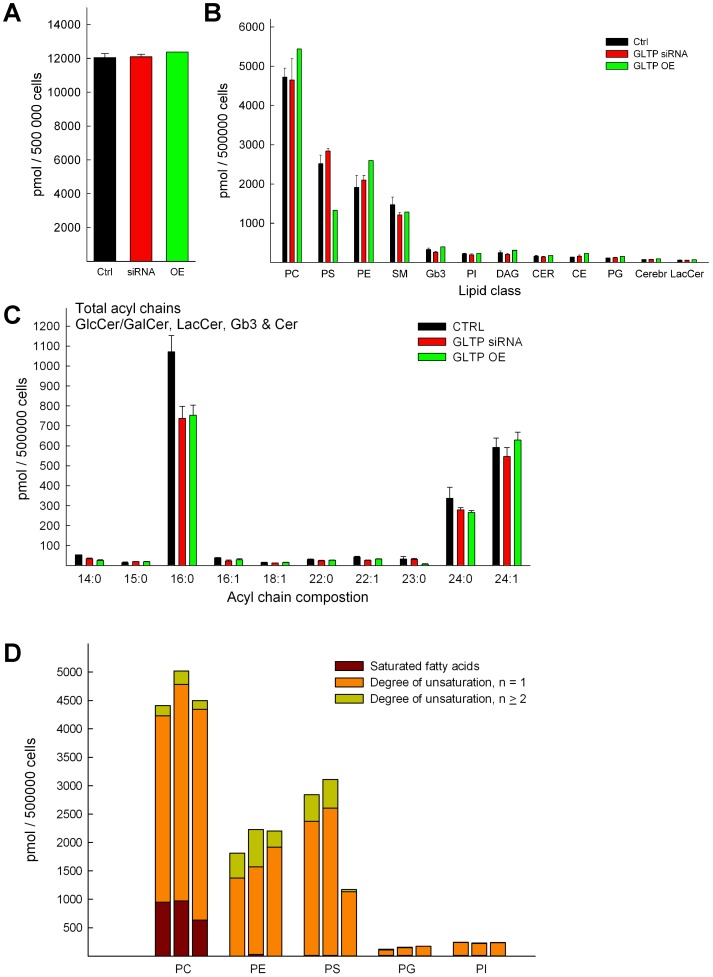
Summarized lipid amounts. (**A**) Comparison of the total lipid content summarized (from the MS data) between CTRL (black bars), GLTP siRNA cells (red bars) and GLTP overexpressing cells (green bars). Lipid quantities were calculated on the basis of the corresponding IS and the amounts (pmol/500000 cells) represented the sum of all individual molecular species of all lipid classes. (**B**) Comparison of the abundance of the lipid classes analyzed in this study. (**C**) Changes in the distribution of the amide-linked hydrocarbon chains in sphingolipids (GlcCer/GalCer, LacCer, Gb_3_, Cer and SM) of normal, GLTP siRNA and GLTP overexpressing HeLa cells. (**D**) Changes in the degree of unsaturation of the acyl chains in the different phospholipid classes relative to GLTP expression. Left control, middle GLTP siRNA and right GLTP OE HeLa cell samples, n is the sum of degree of unsaturation of fatty acid chains in the phospholipids.

The amounts for all 15 different lipid classes were summarized for each HeLa cell type and show a very similar total amount (Fig. 7A). The abundance of the different lipid classes and their changes are summarized in Fig. 7B. Analysis of the distribution of hydrocarbon chains in the sphingolipids of HeLa cells showed that the most abundant acyl chain was palmitic acid (16∶0), followed by the unsaturated nervonic acid (24∶1) and lignoceric acid (24∶0) (Fig. 7C). Interestingly, the amounts of palmitic acid were reduced significantly in both the siRNA and GLTP overexpressing cells. Odd-carbon, especially 15∶0 and 23∶0 carbon chains were also found in the sphingolipids of HeLa cells, but with minor amounts (Fig. 7C). Changes in the saturated and the degree of unsaturation for the phospholipid species are presented in Fig. 5D. PS with fatty acids with 1 unsaturation are much lower and it appears that PS with 2 or more double bonds in their fatty acids are almost lost in HeLa cells overexpressing GLTP (Fig. 7D). There is also a loss of saturated fatty acids in PC for the GLTP overexpressing cells. The fatty acids with two or more double bonds in PE is also less in GLTP overexpressing cells, and elevated in the GLTP siRNA cells.

## Discussion

We present for the first time a comprehensive lipid analysis of 15 different lipid classes, and how they are affected by the GLTP gene down- or up-regulation. We discuss the changes in the lipidome that we observe and how these might relate to GLTP. In particular, a completely new finding is that the globotriaosylceramide Gb_3_ is significantly lower when GLTP is knocked down. Upon up-regulation of GLTP a significant increase in the Gb_3_ levels is also observed. A similar observation was recently found by D’Angelo and coworkers for down-regulating the other member in the GLTP family, FAPP2 [16]. FAPP2 depletion selectively inhibited the synthesis of Gb_3_. FAPP2 down-regulation did not decrease the synthesis of gangliosides GM_3_. In this study, and with this approach, we have not analyzed the changes in the ganglioside families after alternation in the GLTP expressions.

The glycolipid transfer protein family members GLTP and FAPP2 have been connected to many cellular lipid events, and logically the sphingolipid classes have been in focus. Here we present a much broader analysis of the HeLa cell lipidome, covering also membrane phospholipids, cholesterol-esters and diacylglycerols. The compositional changes of 142 lipid molecular species of HeLa cells were analyzed commercially (Zora Biosciences) using shotgun lipidomics. The changes observed in the MS analysis were well in agreement with the metabolic labeling and thin layer chromatography analysis that initiated this study. It should be mentioned that the metabolic labeling and TLC analysis determine the amount of all species in the lipid class, whereas the MS analysis has a cut-off limit and does not always determine the amounts of all acyl chain specific species within some of the lipid classes, especially those with more complex and minor acyl chain components.

When we analyzed the impact of down-regulation of GLTP on the synthesis of GSLs in HeLa cells we found that Gb_3_ decreases in the MS analysis with 25% and almost 25% in the ^3^H-sphinganine metabolic labeling experiments. The up-regulation of GLTP on the other hand, caused an increase in the synthesis of Gb_3_ with about 20% in the MS analysis and almost 40% using ^3^H-sphinganine metabolic labeling. Total GlcCer levels were higher in GLTP overexpressing cells, with GalCer remaining unchanged. A small increase seems to be present in the overexpressing HeLa cells, however not significant. A knockdown of GLTP also showed a somewhat lower quantity of LacCer. Clearly the situation with the expression of GLTP and the LacCer levels needs to be investigated further. D’Angelo and co-workers reported recently that the silencing of FAPP2 caused a dramatic drop in the LacCer and Gb_3_ levels but not in the GM_3_ synthesis in HeLa cells [16]. If the LacCer synthesis is silenced, the synthesis of all three GSLs is inhibited. These results indicate that there are at least two different pools of LacCer in the Golgi apparatus, one for gangliosides, and one for globosides, and that the lactosylceramide synthase is localize both to the Golgi cisternae and to the trans-Golgi network [16]. Previous reports from the Lingwood laboratory also suggested that neutral and acid glycosphingolipids are synthesized from distinct precursor glycosphingolipid pools [36]. These results further indicated that the precursor, GlcCer is transported to the LacCer synthase for globoside synthesis by FAPP2, and that GlcCer destined for LacCer synthase in the early Golgi is transported by other mechanisms, probably vesicular trafficking [5]. Globotriaosylceramide, ceramide trihexoside is also known as CD77 and is a cluster of differentiation. Lactosylceramide 4-alpha-galactosyltransferase (Gb_3_ synthase) is the type II membrane protein that adds an additional galactose to lactosylceramide in the late Golgi to generate Gb_3_ [37].

How can GLTP be linked to changes in Gb_3_ levels? When GLTP is overexpressed more GlcCer for Gb_3_ synthesis is generated. A close examination of our data indicated that the LacCer levels are also somewhat higher than the controls, though not significantly, this both for the MS data and the metabolic labeling experiments (Fig. 1B & Fig. 6A). Speculatively, increased levels of Gb_3_ could be a consequence of a higher activity of GLTP in the ER/early Golgi in directing or sensing more GlcCer for FAPP2, followed by an increased movement of GlcCer to the trans-Golgi. A lower GLTP activity, as in the GLTP siRNA HeLa cells show less Gb_3_, analogously less GlcCer would be presented to FAPP2, and less GlcCer would arrive at the trans-Golgi to be converted to LacCer and subsequently to Gb_3_. A higher GlcCer in GLTP overexpressing cells could be a consequence of the elevated synthesis of Gb_3_, to compensate for maintained levels of other higher GSLs, such as gangliosides, that do not depend on FAPP2 GlcCer transfer activity. For Cer, we only detected lower levels, in GLTP over-expressing cells, for the d18∶0 bases, whereas the d18∶1 bases and total Cer remained at the same level as the controls. However, a strong decrease in total SM is seen in both down- and up-regulated HeLa cells. It appears that the pool of Cer for the altered GlcCer synthesis would come from the regulation of SM synthesis, probably also involving the Cer transporter CERT. There is experimental evidence for an indirect link between GLTP expression to sphingolipid homeostasis through Cer. Brown and colleagues have shown that Cer induced GLTP promoter activity and raised transcript levels (mRNA) in HeLa cells [3]. In HeLa cells, added C_6_-Cer (dissolved in DMSO) altered the in vivo binding affinity of the transcription factors Sp1 and Sp3 for the GLTP promoter and decreased Sp3 acetylation. Whether the externally added short Cers also affect the expression of GLTP on a protein level, is not known.

The sphingolipid homeostasis and its close connection to the cholesterol homeostasis are regulated in a very complicated manner, involving lipid- synthases, sensors and transporters. One common feature for the involved sensors and transporters is that they all are capable of binding to the VAP proteins (VAMP-associated proteins) [38], [39]. The binding to VAPs in the ER occurs through FFAT-like motifs (two phenylalanines in an acidic tract) [40]. The VAPs are highly conserved ER trans-membrane proteins involved in diverse functions. They regulate lipid transport and homeostasis, vesicular trafficking, and are involved in the unfolded protein response [39]. It is therefor likely that GLTP with its FFAT-like motif [2], [40], and capacity to bind to VAP proteins, is also involved in the intricate regulation of sphingolipid homeostasis.

The synthesis of SM is mediated by at least two SM synthases, SMS1 and SMS2 [41]. They catalyze the transfer of a phosphocholine group from PC to the hydroxyl group of Cer at C1. This generates SM and DAG. SM synthesis is largely affected and regulated by the activity of CERT [42]. CERTs role is to transport Cer to the trans-Golgi from the ER [43], [44]. CERT has also been shown to bind and transfer DAG to a minor extent [43], [45]. It has also been suggested that DAG is transported back from the Golgi to ER by CERT [45]. The changes that we see in the DAG levels as a function of GLTP up- and down-regulation, could be a response caused by DAGs ability to be bound by CERT and its VAP association. Likewise, the response in SM levels could also be caused by CERTs activity with VAP. Changes in the Cer and SM as well cholesterol metabolism in the cell is sensed not only by CERT but also by the oxysterol binding protein, OSBP [46]. OSBP, CERT and yet Nir2, a PI/PC transfer protein, all act in concert, to maintain DAG levels in the Golgi [47]–[49]. In the Golgi, protein kinase D associates with the membrane via DAG, and directly phosphorylates CERT, inhibiting its Cer transport activity [50]. Protein kinase D phosphorylation of OSBP results in Golgi fragmentation, that inhibits CERT binding to Golgi membranes [51]. Dephosphorylation increases the Cer transfer mediated by CERT [50], [52].

In cells overexpressing GLTP we detected a significant decrease in PS expression as well as an increase in the CE levels. The synthesis of PS in mammalian cells takes place in the ER, by a base-exchange reaction, where PS synthase-1 (PSS-1) primarily uses PC as a substrate and PS synthase-2 (PSS-2), PE as the base for exchange with serine [53]. Neither PC nor PE changed significantly, regardless of GLTP expression, despite the large decrease in PS levels. Both PSS-1 and PSS-2 are integral ER membrane proteins sub-compartmentalized to the mitochondria-associated membranes, MAM fractions. MAMs are a region of ER closely associated with the mitochondria. PSS-1 and PSS-2 do not seem to have an FFAT-like domain and have to our knowledge not been reported to bind to the VAP-proteins. The enzymatic activities required for synthesis of triacylglycerols, CE, and free cholesterol have also been located to the MAM fractions [54]. In addition, the synthesis of GlcCer has been reported to occur also in the MAMs [55] and not only in the cis-Golgi [56], [57]. Furthermore, Meyer and de Groot suggested that PS might serve as the serine donor in the initial condensation of serine and palmitoyl coenzyme A catalyzed by serine palmitoyltransferase, the initial step in Cer biosynthesis [58]. This metabolic pathway together with the lipid metabolism in the MAMs would connect PS and CE to the Cer and GlcCer biosynthesis and could as discussed above be impacted by the expression of GLTP and its capacity to bind to the VAP-proteins. In a previous study Gao and coworkers speculate that overexpression of GLTP in HeLa cells would limit the availability of GlcCer for generation of higher GSLs, and the net effect would be reduced levels of complex GSLs [4]. GLTP would function as a sink protecting GlcCer from being processed. However, here we show that overexpression rather significantly elevates at least Gb_3_ and to some extent also LacCer, and would rather function as an activator for the synthesis of Gb_3_.

Summarizing, the changed in the lipidome that we observe in this work is likely to be a consequence of GLTPs involvement, as a glycolipid binder, sensor or transporter together with the other VAP protein binding players. In a broader perspective, since different species of Cer are destined for different sphingolipid end products [59]–[62], we speculate that GLTP might play a role in orchestrating the transfer of different GlcCer species to different destinations, with connections to the synthesis of Cer precursors or their transfer by vesicular means or by CERT. Perhaps the GlcCer destined to be transported via FAPP2 to the pool of LacCer specifically destined to Gb_3_ synthesis is sensed/controlled by GLTP. With this work we put forward new data suggesting that GLTP would be a significant player in not only the sphingolipid metabolism but also could have a much broader role in the lipid metabolism in the cell.

## Supporting Information

Figure S1 **Efficiency of small interfering RNA on the GLTP protein expression in HeLa cells at different time intervals.** **(A)** qPCR analysis of GLTP mRNA expression levels (in percent) after treatment with different GLTP siRNA sequences. All three siRNA sequences, termed #76 (red), #77 (green) and #78 (yellow) used in this work were compared and normalized to the scrambled siRNA universal control (UC, black). The three different sequences are described in the Materials and Methods section. **(B)** Western blotting analysis shows a reduced protein expression in HeLa siRNA transfected cells compared both the control and the UC samples. The GLTP expression was analyzed at different time intervals, after siRNA treatment. Immunoblot against human GLTP (upper blot) in normal HeLa cells, UC control cells and different GLTP siRNA sequences, 50 µg of total cell lysates were used and a rabbit anti-GLTP antibody.
(TIFF)

Figure S2 **Efficiency of overexpression on the GLTP protein levels in HeLa cells.** **(A)** The upper blot shows the beta-actin expression and lower blot the expression of GLTP as a function of time. All lanes are loaded with 30 µg total protein whole cell lysate. Note the endogenous expression of GLTP is low and not visible in the lower blot, first lane. **(B)** Western blot analysis of the expression of GLTP in the HeLa cells used for the MS lipidomics analysis. Left blot shows the endogenous expression of GLTP (lane 1) and the reduced protein expression in HeLa cells with GLTP knockdown, by siRNA (#77 siRNA GLTP gene construct), lane 2. A total of 80 µg total cell lysate was loaded, and beta-actin was used as the loading control, upper blot. The right blot shows the amount of GLTP in HeLa cells with GLTP overexpression (lane 4), and an invisible endogenous GLTP band in lane 3, due to the loading amount of just 10 µg total cell lysate.
(TIFF)

Table S1 The amounts for the molecular lipids in HeLa cells are presented as pmol/500000 cells for CTRL cells down- (GLTP siRNA) and up-regulated (GLTP OE) cell samples. The values for the CTRL are averages of thee mock samples, GLTP siRNA averages of two samples, and GLTP OE values from one sample.
(DOCX)

Table S2 The amounts for the molecular lipids in HeLa cells are presented as pmol/500000 cells for CTRL cells down- (GLTP siRNA) and up-regulated (GLTP OE) cell samples. The values for the CTRL are averages of thee mock samples, GLTP siRNA averages of two samples, and GLTP OE values from one sample. Blank values means that the concentration is below the quantification limit or a value is not included in the final data set because of quality control (QC, see Materials and Methods) cutoff.
(DOCX)

Table S3 The amounts for the molecular lipids in HeLa cells are presented as pmol/500000 cells for CTRL cells down- (GLTP siRNA) and up-regulated (GLTP OE) cell samples. The values for the CTRL are averages of thee mock samples, GLTP siRNA averages of two samples, and GLTP OE values from one sample. Blank values means that the concentration is below the quantification limit or a value is not included in the final data set because of quality control (QC, see Materials and Methods) cutoff.
(DOCX)

Table S4 The amounts for the molecular lipids in HeLa cells are presented as pmol/500000 cells for CTRL cells down- (GLTP siRNA) and up-regulated (GLTP OE) cell samples. The values for the CTRL are averages of thee mock samples, GLTP siRNA averages of two samples, and GLTP OE values from one sample. Blank values means that the concentration is below the quantification limit or a value is not included in the final data set because of quality control (QC, see Materials and Methods) cutoff.
(DOCX)
