# Supplementary figures and images for: Alternation in the Glycolipid Transfer Protein Expression Causes Changes in the Cellular Lipidome
Source: PLoS One. 2014 May 13;9(5):e97263. doi: 10.1371/journal.pone.0097263 (PMC4019525; doi:10.1371/journal.pone.0097263)

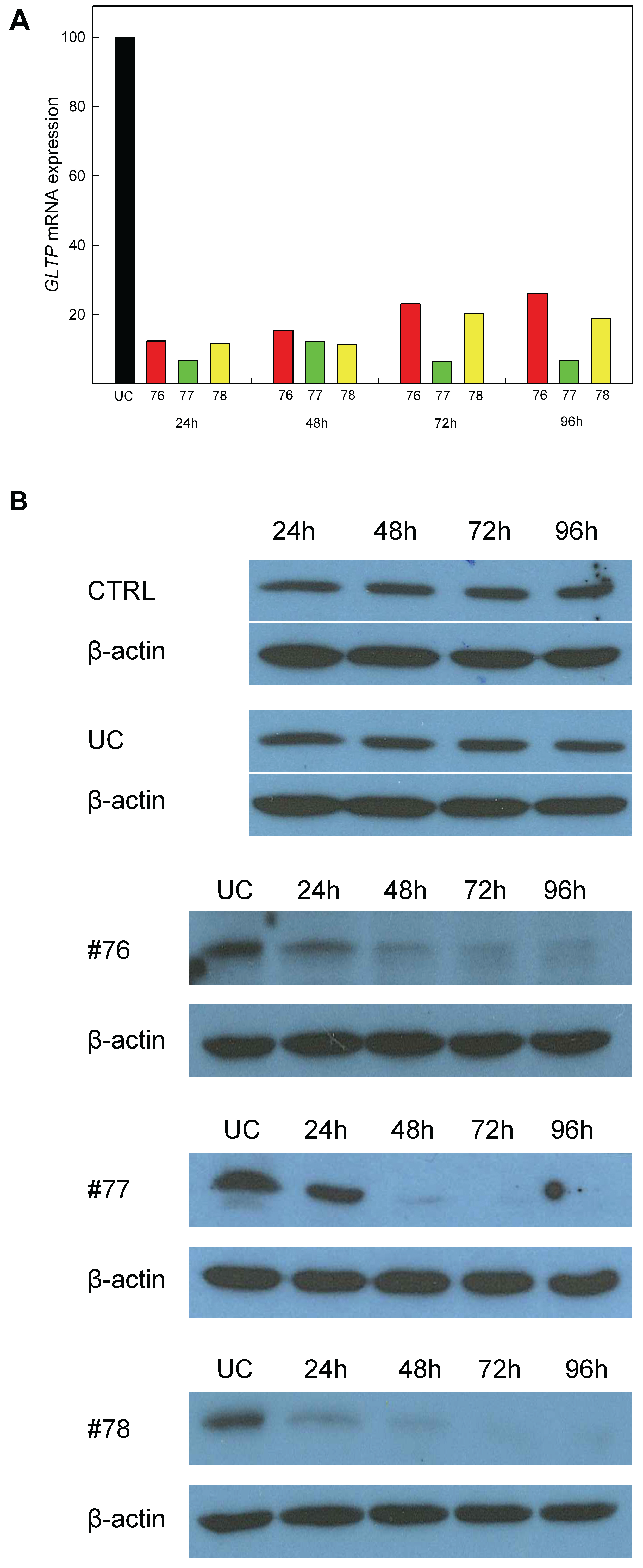

Supplement: Figure S1 — Efficiency of small interfering RNA on the GLTP protein expression in HeLa cells at different time intervals. (A) qPCR analysis of GLTP mRNA expression levels (in percent) after treatment with different GLTP siRNA sequences. All three siRNA sequences, termed #76 (red), #77 (green) and #78 (yellow) used in this work were compared and normalized to the scrambled siRNA universal control (UC, black). The three different sequences are described in the Materials and Methods section. (B) Western blotting analysis shows a reduced protein expression in HeLa siRNA transfected cells compared both the control and the UC samples. The GLTP expression was analyzed at different time intervals, after siRNA treatment. Immunoblot against human GLTP (upper blot) in normal HeLa cells, UC control cells and different GLTP siRNA sequences, 50 µg of total cell lysates were used and a rabbit anti-GLTP antibody. (TIFF) [file pone.0097263.s001.tiff]

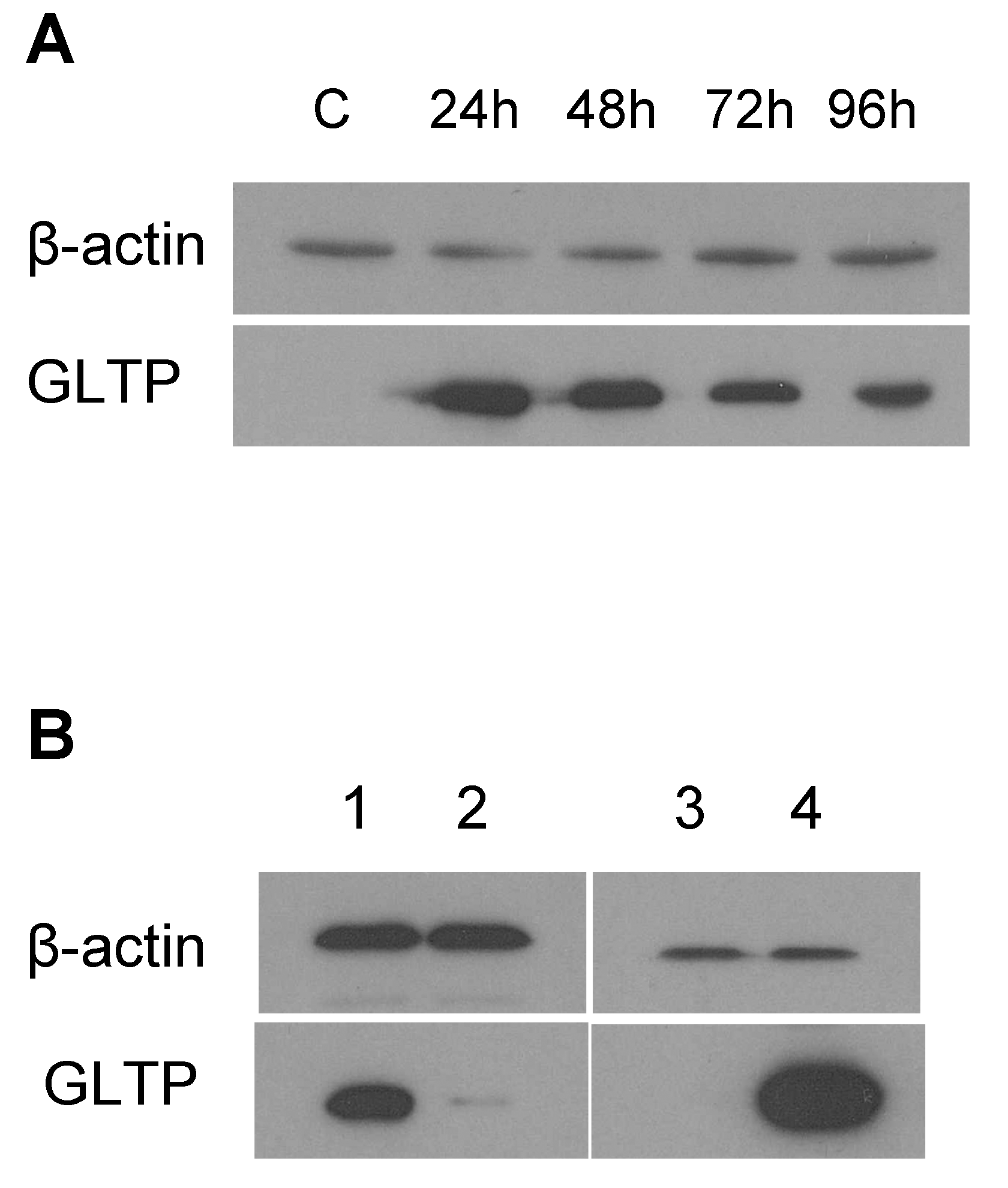

Supplement: Figure S2 — Efficiency of overexpression on the GLTP protein levels in HeLa cells. (A) The upper blot shows the beta-actin expression and lower blot the expression of GLTP as a function of time. All lanes are loaded with 30 µg total protein whole cell lysate. Note the endogenous expression of GLTP is low and not visible in the lower blot, first lane. (B) Western blot analysis of the expression of GLTP in the HeLa cells used for the MS lipidomics analysis. Left blot shows the endogenous expression of GLTP (lane 1) and the reduced protein expression in HeLa cells with GLTP knockdown, by siRNA (#77 siRNA GLTP gene construct), lane 2. A total of 80 µg total cell lysate was loaded, and beta-actin was used as the loading control, upper blot. The right blot shows the amount of GLTP in HeLa cells with GLTP overexpression (lane 4), and an invisible endogenous GLTP band in lane 3, due to the loading amount of just 10 µg total cell lysate. (TIFF) [file pone.0097263.s002.tif]
