# Supplementary material for: Alternation in the Glycolipid Transfer Protein Expression Causes Changes in the Cellular Lipidome
Source: PLoS One. 2014 May 13;9(5):e97263. doi: 10.1371/journal.pone.0097263 (PMC4019525; doi:10.1371/journal.pone.0097263)
Supplement: Table S1 — The amounts for the molecular lipids in HeLa cells are presented as pmol/500000 cells for CTRL cells down- (GLTP siRNA) and up-regulated (GLTP OE) cell samples. The values for the CTRL are averages of thee mock samples, GLTP siRNA averages of two samples, and GLTP OE values from one sample. (DOCX) [file pone.0097263.s003.docx]

| Lipid Class | Acyl chain | CTRL |  |  | GLTP siRNA |  |  | GLTP OE |
| --- | --- | --- | --- | --- | --- | --- | --- | --- |
| GlcCer/GalCer | d18:0/16:0 | 19.2 | ±1.5 |  | 17.9 | ±1.0 |  | 23.6 |
|  | d18:0/18:0 | 4.7 | ±0.8 |  | 4.6 | ±0.1 |  | 7.1 |
|  | d18:0/24:0 | 0.4 | ±0.1 |  | 0.4 | ±0.1 |  | 0.3 |
|  | d18:0/24:1 | 0.4 | ±0.1 |  | 0.4 | ±0.1 |  | 0.5 |
|  | *Total, d18:0 base* | *24.8* | ±*2.0* |  | *23.6* | ±*1.2* |  | *31.6* |
|  | d18:1/16:0 | 11.2 | ±0.9 |  | 10.8 | ±0.9 |  | 13.7 |
|  | d18:1/18:0 | 0.6 | ±0.2 |  | 0.6 | ±0.0 |  | 0.6 |
|  | d18:1/20:0 | 0.5 | ±0.1 |  | 0.4 | ±0.1 |  | 0.4 |
|  | d18:1/22:0 | 6.0 | ±1.2 |  | 6.1 | ±0.4 |  | 5.0 |
|  | d18:1/24:0 | 16.7 | ±1.2 |  | 16.7 | ±1.1 |  | 17.1 |
|  | d18:1/24:1 | 17.0 | ±1.3 |  | 20.3 | ±2.1 |  | 21.3 |
|  | d18:1/26:1 | 0.8 | ±0.1 |  | 0.8 | ±0.1 |  | 2.0 |
|  | *Total, d18:1 base* | *52.9* | ±*2.7* |  | *55.8* | ±*4.5* |  | *59.2* |
|  | **Total GlcCer/GalCer** | **77.7** | **±3.9** |  | **79.5** | **±5.7** |  | **90.8** |
| LacCer | d18:0/16:0 | 2.3 | ±0.1 |  | ±2.1 | 0.1 |  | 2.3 |
|  | d18:0/24:0 | 0.3 | ±0.1 |  | ±0.1 | 0.0 |  | 0.3 |
|  | d18:0/24:1 | 0.4 | ±0.1 |  | ±0.5 | 0.1 |  | 0.6 |
|  | *Total, d18:0 base* | *3.3* | *±0.3* |  | *±2.9* | *0.2* |  | *3.3* |
|  | d18:1/16:0 | 23.9 | ±3.1 |  | 23.1 | ±2.0 |  | 29.0 |
|  | d18:1/18:0 | 2.3 | ±0.3 |  | 2.0 | ±0.2 |  | 2.4 |
|  | d18:1/20:0 | 0.5 | ±0.1 |  | 0.5 | ±0.1 |  | 0.7 |
|  | d18:1/22:0 | 4.9 | ±0.5 |  | 4.8 | ±0.5 |  | 6.4 |
|  | d18:1/24:0 | 10.0 | ±1.8 |  | 8.7 | ±1.0 |  | 12.0 |
|  | d18:1/24:1 | 13.7 | ±0.7 |  | 13.7 | ±0.9 |  | 18.2 |
|  | d18:1/26:1 | 0.6 | ±0.1 |  | 0.6 | ±0.1 |  | 0.6 |
|  | *Total, d18:1 base* | *56.0* | ±*6.5* |  | *53.6* | ±*4.9* |  | *69.4* |
|  | **Total LacCer** | **59.2** | **±6.7** |  | **56.5** | **±5.1** |  | **72.8** |
| Cer | d18:0/16:0 | 4.0 | ±1.0 |  | 2.8 | ±0.2 |  | 2.1 |
|  | d18:0/18:0 | 0.4 | ±0.1 |  | 0.3 | ±0.1 |  | 0.2 |
|  | d18:0/22:0 | 2.2 | ±0.2 |  | 1.3 | ±0.1 |  | 0.5 |
|  | d18:0/24:0 | 0.9 | ±0.1 |  | 0.8 | ±0.1 |  | 0.9 |
|  | d18:0/24:1 | 4.8 | ±0.4 |  | 3.9 | ±0.3 |  | 1.6 |
|  | *Total, d18:0 base* | *12.5* | *±0.8* |  | *9.4* | *±0.7* |  | *5.3* |
|  | d18:1/16:0 | 35.3 | ±3.1 |  | 28.3 | ±2.2 |  | 39.8 |
|  | d18:1/18:0 | 4.8 | ±1.1 |  | 3.7 | ±0.2 |  | 4.3 |
|  | d18:1/20:0 | 2.1 | ±0.5 |  | 1.7 | ±0.2 |  | 1.8 |
|  | d18:1/22:0 | 14.2 | ±2.2 |  | 13.2 | ±1.1 |  | 13.9 |
|  | d18:1/24:0 | 38.2 | ±3.2 |  | 33.6 | ±1.0 |  | 37.4 |
|  | d18:1/24:1 | 56.2 | ±3.5 |  | 53.6 | ±4.7 |  | 68.8 |
|  | d18:1/26:0 | 1.0 | ±0.1 |  | 0.9 | ±0.1 |  | 1.1 |
|  | d18:1/26:1 | 2.5 | ±0.7 |  | 2.1 | ±0.1 |  | 3.3 |
|  | *Total, d18:1 base* | *154.3* | *±9.9* |  | *137.1* | *±9.7* |  | *170.4* |
|  | **Total Cer** | **164.5** | **±12.5** |  | **146.5** | **±10.4** |  | **175.7** |
