# Supplementary material for: Alternation in the Glycolipid Transfer Protein Expression Causes Changes in the Cellular Lipidome
Source: PLoS One. 2014 May 13;9(5):e97263. doi: 10.1371/journal.pone.0097263 (PMC4019525; doi:10.1371/journal.pone.0097263)
Supplement: Table S4 — The amounts for the molecular lipids in HeLa cells are presented as pmol/500000 cells for CTRL cells down- (GLTP siRNA) and up-regulated (GLTP OE) cell samples. The values for the CTRL are averages of thee mock samples, GLTP siRNA averages of two samples, and GLTP OE values from one sample. Blank values means that the concentration is below the quantification limit or a value is not included in the final data set because of quality control (QC, see Materials and Methods) cutoff. (DOCX) [file pone.0097263.s006.docx]

| Lipid Class | Acyl chain | CTRL |  |  | GLTP siRNA |  |  | GLTP OE |
| --- | --- | --- | --- | --- | --- | --- | --- | --- |
| PE | 16:0-16:1 | 69.6 | ±16.9 |  | 66.5 | ±2.7 |  | 80.62 |
|  | 16:0-18:1 | 268.2 | ±56.1 |  | 255.6 | ±5.8 |  | 424.0 |
|  | 16:0-20:4 | 30.8 | ±4.7 |  | 39.0 | ±2.4 |  | 32.3 |
|  | 16:1-18:0 | 123.2 | ±19.7 |  | 130.7 | ±3.2 |  | 137.5 |
|  | 16:1-18:1 | 162.7 | ±33.9 |  | 179.3 | ±14.6 |  | 205.8 |
|  | 18:0-18:1 | 353.3 | ±37.5 |  | 346.5 | ±23.5 |  | 438.7 |
|  | 18:0-18:2 | 41.3 | ±1.9 |  | 42.1 | ±4.8 |  | 43.6 |
|  | 18:0-20:3 | 38.5 | ±1.1 |  | 40.4 | - |  | 58.7 |
|  | 18:0-20:4 | 174.5 | ±58.3 |  | 196.6 | ±15.4 |  | 147.1 |
|  | 18:1-18:1 | 472.4 | ±35.1 |  | 457.5 | ±19.3 |  | 687.6 |
|  | 18:1-18:2 | 63.9 | ±3.9 |  | 62.8 | ±2.0 |  | 69.8 |
|  | 18:1-20:1 | 110.9 | ±11.0 |  | 104.9 | ±13.1 |  | 120.5 |
|  | 18:1-20:3 | 52.2 | ±1.8 |  | 45.4 | ±0.1 |  | 39.6 |
|  | 18:1-20:4 | 73.6 | ±15.0 |  | 79.6 | ±3.8 |  | 82.2 |
|  | **Total PE** | **2107.1** | **±19.0** |  | **2046.6** | **±115.9** |  | **2568.1** |
| PE O | O-18:0/16:0 | 19.0 | ±0.1 |  | 22.4 | - |  | 23.4 |
|  | O-18:0/18:1 | 30.0 | ±2.3 |  | 22.7 | ±4.3 |  | 30.2 |
|  | **Total PE O** | **49.0** | **±2.4** |  | **49.3** | **±2.3** |  | **53.6** |
| PS | 16:0-16:1 | 113.7 | ±9.5 |  | 137.3 | ±14.4 |  | 63.3 |
|  | 16:0-18:1 | 335.6 | ±28.3 |  | 317.4 | ±10.3 |  | 181.5 |
|  | 16:1-18:0 | 275.9 | ±32.7 |  | 330.9 | ±11.5 |  | 135.9 |
|  | 16:1-18:1 | 131.4 | ±27.0 |  | 127.1 | ±1.8 |  | 59.5 |
|  | 18:0-18:1 | 1293.9 | ±27.7 |  | 1254.9 | ±50.5 |  | 615.1 |
|  | 18:0-18:2 | 101.9 | ±21.1 |  | 107.8 | ±7.4 |  | 36.8 |
|  | 18:0-20:3 | 84.6 | - |  | 69.2 | ±5.7 |  | 24.0 |
|  | 18:0-20:4 | 186.4 | ±18.7 |  | 117.1 | ±5.2 |  | - |
|  | 18:0-22:5 | 126.6 | ±19.2 |  | 105.9 | ±4.3 |  | 23.4 |
|  | 18:1-18:1 | 329.9 | ±49.2 |  | 365.0 | ±1.5 |  | 167.6 |
|  | 18:1-20:1 | 54.0 | - |  | 46.7 | - |  | 24.6 |
|  | **Total PS** | **2805.3** | **±179.7** |  | **3226.6** | **±40.25** |  | **1331.6** |
| PI | 16:0-18:1 | 85.4 | ±9.8 |  | 70.3 | ±7.9 |  | 84.3 |
|  | 18:0-18:1 | 69.2 | ±7.1 |  | 61.6 | ±7.6 |  | 77.7 |
|  | 18:1-18:1 | 66.4 | ±0.9 |  | 63.9 | ±7.4 |  | 69.9 |
|  | **Total PI** | **220.8** | **±16.2** |  | **195.8** | **±22.9** |  | **231.9** |
| PG | 16:0-18:1 | 66.3 | ±2.9 |  | 72.6 | ±4.1 |  | 96.6 |
|  | 18:1-18:1 | 40.0 | ±8.1 |  | 50.8 | ±5.4 |  | 59.7 |
|  | **Total PG** | **113.1** | **±646** |  | **123.4** | **±9.3** |  | **156.3** |
